# Non-Invasive Spinal Cord Stimulation for Motor Rehabilitation of Patients with Spinal Muscular Atrophy Treated with Orphan Drugs

**DOI:** 10.3390/biomedicines12061162

**Published:** 2024-05-24

**Authors:** Anton Novikov, Maria Maldova, Natalia Shamantseva, Ivan Shalmiev, Elena Shoshina, Natalia Epoyan, Natalia Krutikova, Tatiana Moshonkina

**Affiliations:** 1 EirMED, 10 Vsevolod Vishnevsky St., 197136 St. Petersburg, Russia; 2 Pavlov Institute of Physiology, Russian Academy of Sciences, 6 Makarova Enb., 199034 St. Petersburg, Russia

**Keywords:** spinal muscular atrophy, spinal cord stimulation, physical therapy, nusinersen, risdiplam, onasemnogene abeparvovec

## Abstract

Spinal muscular atrophy (SMA) is an orphan disease characterized by the progressive degeneration of spinal alpha motor neurons. In recent years, nusinersen and several other drugs have been approved for the treatment of this disease. Transcutaneous spinal cord stimulation (tSCS) modulates spinal neuronal networks, resulting in changes in locomotion and posture in patients with severe spinal cord injury and stroke. We hypothesize that tSCS can activate motor neurons that are intact and restored by medication, slow the decline in motor activity, and contribute to the development of motor skills in SMA patients. Thirty-seven children and adults with SMA types 2 and 3 participated in this study. The median duration of drug treatment was over 20 months. The application of tSCS was performed during physical therapy for 20–40 min per day for ~12 days. Outcome measures were specific SMA motor scales, goniometry of contractured joints, and forced vital capacity. Significant increases in motor function, improved respiratory function, and decreased contracture were observed in both type 2 and 3 SMA participants. The magnitude of functional changes was not associated with participant age. Further studies are needed to elucidate the reasons for the beneficial effects of spinal cord electrical stimulation on SMA.

## 1. Introduction

Spinal muscular atrophy (SMA) is a genetic disease characterized by progressive muscle weakness caused by spinal motor neuron dysfunction. Rehabilitation of patients with SMA aims to slow the loss of motor skills and reduce the burden of the disease. Since 2016, orphan drugs (nusinersen and others) have been used in clinical practice to correct the genes that are directly related to the pathogenesis of SMA. Previously, we demonstrated in a case series that transcutaneous spinal cord stimulation (tSCS) combined with physical therapy is an effective rehabilitation method for patients treated with nusinersen [1]. We now evaluated the effect of tSCS in 37 SMA patients treated with nusinersen and other orphan drugs and investigated the correlation between SMA severity, patient age, duration of drug therapy, and extent of motor changes after tSCS treatment.

### 1.1. Spinal Muscular Atrophy

SMA is caused by the progressive degeneration of spinal alpha motor neurons, resulting in the progressive weakness and atrophy of proximal muscles [2,3]. Alpha motor neuron degeneration is caused by the deletion or mutation of the SMN1 gene [4]. This gene is responsible for the activity and survival of motor neurons. The paralogous SMN2 gene is similar to the SMN1 gene, except for several single-nucleotide substitutions, including one in exon 7, resulting in aberrant pre-mRNA splicing and exon 7 skipping in nearly 90% of transcripts. The severity of SMA correlates with the copy number of the SMN2 gene carried by patients [2].

There are five SMA phenotypes; they differ in age of onset and maximum achievable motor function associated with that age, and three of them are major phenotypes [5]. SMA type 1 manifests in the first 6 months after birth; maximum motor skills are sitting with support. SMA type 2 manifests between the first 6 and 18 months of life, and patients with type 2 are able to sit independently. The onset of SMA type 3 occurs after 18 months of age; patients are able to stand upright and walk. Patients with SMA type 1 usually have two or three copies of SMN2; SMA type 2 patients have three copies, and SMA type 3 patients have three or four copies [6]. The classification of SMA into five phenotypes is conditional; in practice, these phenotypes represent a continuum. Clinicians identify additional subtypes, and patients with SMA are characterized by functional status as “non-sitters”, “sitters”, or “walkers” [5]. In terms of functional abilities, SMA patients are also defined as non-ambulatory patients who are able to sit independently and as ambulatory patients [5].

### 1.2. SMA Orphan Drugs

SMA is an orphan disease with an incidence of less than 5 per 10,000. Drugs used to treat rare diseases are called orphan drugs [7]. Drugs that correct the function of genes directly involved in the pathogenesis of SMA have entered clinical practice in the last decade [8].

The first disease-modifying drug, nusinersen, was approved for the pharmaceutical market by the U.S. Food and Drug Administration in 2016, and showed good efficacy in the early stages of the disease [9,10]. Nusinersen is an antisense oligonucleotide drug; its activity is based on the correction of the splicing of exon 7 of the endogenous SMN2 pre-mRNA. The drug has no restrictions on age and type of SMA. It is taken for life; maintenance doses are administered intrathecally every 4 months.

Onasemnogene abeparvovec was approved in 2019 for the treatment of patients younger than 2 years of age [11]. It is an adeno-associated virus serotype 9 that carries SMN complementary DNA encoding the missing SMN protein and is administered once intravenously [12]. 

The third entry was risdiplam, which was approved in 2020 for SMA patients older than 2 months [7,13]. It is a pyridine derivative, an oral compound that modifies SMN2 splicing to increase SMN production. This drug is given daily.

The objective of treatment with nusinersen, risdiplam, and onasemnogen abeparvovec is to increase the survival of motor neurons. Comparative studies of the efficacy of these drugs are currently lacking. Consequently, patients treated with any of these orphan drugs specific to SMA were included in this study.

### 1.3. Transcutaneous Spinal Cord Stimulation

In our study, tSCS is electrical stimulation by rectangular pulses at a frequency of ~30 Hz modulated with 5–10 kHz pulses. The electrodes were placed on the skin—cathodes over the spinal motor centers, at the level of the C3–C5, T11–T12, or L1–L2 vertebrae, and anodes over the iliac crest or clavicles [1]. 

The potential neural structures that may be activated during tSCS were clearly demonstrated in [14]. Low-current stimulation primarily engages low-threshold Ia large-diameter afferents and is accompanied by the activation of locomotor spinal networks and further involvement of motor axons. As stimulation intensity increases, smaller-diameter afferents (group Ib, cutaneous afferents, group II muscle spindle afferents) and an even greater number of intraspinal connections and spinal interneurons are activated. In studies conducted on animal models and healthy volunteers, it has been demonstrated that this modulation of multiple pathways connecting multiple sensory afferent types to interneurons also receives supraspinal input, which generates the complex coordination of multiple motor pools. Furthermore, direct motor activation also occurs with high stimulation current. Subsequent studies have validated this concept. Many studies with healthy subjects have shown that tSCS modulates spinal neuronal networks, manifested by changes in locomotion and posture [15,16,17]. 

This non-invasive spinal cord stimulation has a clinical impact [18,19,20]. In patients paralyzed by spinal cord injury, tSCS induces and improves voluntary movement and muscle strength and function [18,20,21]. In stroke patients, significant and clinically meaningful differences in walking parameters were achieved after two weeks of tSCS in combination with physical therapy [22]. In children with cerebral palsy, a similar short course of rehabilitation with tSCS resulted in significant and meaningful clinical differences in Gross Motor Function Measure scores [23,24], and acute tSCS improved postural and locomotor skills [25]. 

### 1.4. Purpose and Hypothesis of This Study

In all of the above cases where tSCS was used to activate spinal motor centers located in “healthy” areas of the spinal cord, the motor deficits were caused by injury to brain–muscle signaling or abnormal brain activity. In SMA, the alpha motor neurons of the spinal cord are genetically affected, resulting in progressive muscle weakness and the development of muscle atrophy. It has been shown that 18 months of nusinersen treatment in children dramatically increases the number of motor neurons [26], and 10 months of risdiplam treatment in adult patients increases the active motor unit pool [27].

We hypothesized that in patients with SMA, tSCS could activate intact motor neurons and motor neurons that had been restored after treatment with SMA-specific drugs, leading to a slower decline in motor activity and the development of motor skills. We obtained encouraging results with stimulation in five patients with SMA types 2 and 3 [1]. In this article, we verify these results in a study with a larger number of patients and also examine the efficacy of tSCS in relation to SMA type, functional status, age, and duration of medication.

## 2. Materials and Methods

This study was performed at the EirMED Rehabilitation Center (St. Petersburg, Russia) between November 2022 and November 2023. Procedures, training, and outcome measures were performed in accordance with the Declaration of Helsinki and approved by the Ethics Committee of EirMED Rehabilitation Center (#22-01, approved on 12 August 2022). 

The inclusion criteria were as follows: a genetically and clinically confirmed diagnosis of SMA type 2 or 3; the administration of any SMA-specific orphan drug; and the absence of decompensated somatic pathology. The exclusion criteria included an inability to comprehend and fulfill the instructions of the trainers and investigators, damage to or inflammation of the skin where the stimulating electrodes are attached, respiratory compromise that would compromise the safety of travel to the treatment site, and participant location that was deemed to be beyond a reasonable driving distance by the investigators. Written informed consent was obtained from all adult participants and parents of child participants.

### 2.1. Participants

Thirty-seven participants with genetically confirmed SMA type 2 or 3 were studied (Table 1, Tables S1 and S2). Thirty participants were treated with nusinersen, six with risdiplam, and one with onasemnogene abeparvovec. Treatment with orphan drugs was continued for 23 ± 9 and 28 ± 12 months in the SMA type 2 and 3 groups, respectively. Participants had previously received physical therapy aimed at preventing the loss of muscle strength and the development of joint contractures.

### 2.2. Procedures

The stimulation of the spinal cord was performed in combination with physical therapy. These interventions aimed to achieve personalized treatment goals. Detailed examples of personalized treatment goals, physical exercises, and the tSCS strategy for five SMA type 2 and 3 patients can be found in our previous study [1]. Physical therapy with tSCS took ~1 h per day and was carried out 6 days a week. Moreover, physical procedures such as massages and balneotherapy were performed to improve coordination and proprioception. In addition, there were sessions with an ergo therapist and a speech therapist. The intensity of those sessions was adapted to the individual constitution of the participants. All therapies, including tSCS training and other procedures, lasted 3 h per day. 

Participants came to the procedures with caregivers (family members). In cases where caregivers were unable to accompany the participant for 12 procedures and 2 days of outcome testing, a reduction to 10 procedures was allowed. The total duration of therapy was 12 ± 0.8 and 12 ± 0.7 days in SMA type 2 and 3, respectively (Tables S3 and S4).

#### 2.2.1. Treatment Goals

Treatment goals were individualized and determined using the SMART (specific, measurable, achievable, realistic/relevant, and timed) method because this is an effective method to achieve behavioral change and enhance the quality of life [28]. Individual goals ranged from “turning from supine to lateral position, increasing hand strength, increasing endurance, decreasing joint contracture” (participant 7K14) to “improving stepping pattern, increasing walking speed” (participant 15K48).

#### 2.2.2. Physical Therapy

Physical exercises included passive and active stretching movements in the joints of the upper and lower extremities, passive and active positioning, moving and holding weights, body movements to prevent scoliosis, stepping and kicking movements, breathing exercises, etc. In some cases, to facilitate limb movements, the movements were performed with the limb in a gravity-neutral position, with the participant lying on their side, with the leg or arm supported by a swing [14,29].

#### 2.2.3. Transcutaneous Spinal Cord Stimulation

One, two, or three regions of the spinal cord above the cervical and/or lumbar enlargements of the spinal cord and/or above the sacral segments were stimulated (Tables S3 and S4). When intended to target the muscles of the upper extremities, stimulation of the neck and upper thoracic region was used. Stimulation of the lower thoracic, lumbar, and coccygeal regions was performed to influence the muscles of the trunk and lower extremities. Stimulation sites were selected depending on rehabilitation goals considering the results of all previously published studies [18,30,31,32].

Two adhesive round electrodes (Ø 2.5 cm; ValuTrode^®^ Axelgaard Manufacturing Co., Fallbrook, CA, USA) were placed on the skin above the spinous process of either the C5, Th11, L1, and L5 vertebrae or the coccyx. These electrodes were independent cathodes. Two adhesive rectangular electrodes (4 × 5 cm^2^; ValuTrode^®^ Axelgaard Manufacturing Co., Fallbrook, CA, USA) placed above the iliac crest were common anodes. Bipolar rectangular pulses of 1 ms duration, ~30 Hz, modulated at 5 kHz, produced by Neostim-5 (Cosyma Ltd., Moscow, Russia) were used.

The intensity of tSCS was determined individually for each participant so that the current amplitude was maximally tolerable without causing unpleasant sensations. Intensity was adjusted by the therapists from session to session to follow this rule.

Stimulation, excluding breaks to relax participants and change positions for the next therapeutic exercise, is presented in Tables S3 and S4. 

### 2.3. Outcome Measures

Tests were performed one day before and one day after the treatment course in the absence of tSCS. Outcome measures were specific to SMA patients [33,34]. From the spectrum of all specific tests, invasive tests were selected to assess disability and functional limitations specific to both type 2 and type 3 SMA patients. Efficacy criteria were contracture joint goniometry [35], Revised Upper Limb Module (RULM) [36], modified Hammersmith Function Motor Scale Expanded (HFMSE) [37], and forced vital capacity (FVC) [38]. Participants underwent all the tests that they were able to perform according to their physical abilities.

Therapists documented each session. Information on tSCS details, changes in physical exercises, and possible side effects or adverse events was available from session to session.

Therapists, adult participants, and parents of child participants were interviewed on the last day of the course of treatment about significant changes in motor activity and motor skills that they observed during the course of this study. 

#### 2.3.1. Joint Goniometry

The ranges of passive motion of the shoulder, elbow, wrist, hip, knee, and ankle were determined and documented for each participant. Knee contracture was the most common contracture of the joints in the participant population. Changes in knee range of motion (ROM) were analyzed in the SMA type 2 and 3 groups. Knee extension was measured in the supine position with the hip extended as well as with the hip flexed to 90 degrees. According to the standards [39], a single determination of ROM was performed. 

#### 2.3.2. Revised Upper Limb Module

The RULM scale is a robust clinical measure for assessing upper limb motor function in SMA [36]. The scale establishes functional levels covering distal to proximal movements. It is a 20-item scale with a maximum score of 37, with higher scores indicating better upper limb function.

#### 2.3.3. Hammersmith Function Motor Scale Expanded

The HFMSE is a standard scale of functional ability in patients with SMA types 2 and 3 [37]. This scale comprises 33 items; the maximum possible score is 66. A limitation of this test is the inability to sit on a chair, couch, or floor (not in a wheelchair) for ~3 s without back and hand support.

#### 2.3.4. Forced Vital Capacity

FVC appears to be the most reliable measure among other pulmonary parameters to be used as outcomes in SMA [33,40]. According to the guidelines [38], absolute values of FVC were determined three times. During the maneuver, participants were asked to inhale rapidly until completely full, then exhale with maximum effort until completely empty and then inhale with maximum effort until completely full. Participants were required to exert significant physical effort while performing the maneuver. The test was completed by participants who were able to perform three FVC maneuvers to standard. The largest FVC observed from three of the acceptable values was compared with age-matched controls as a percentage predicted for age, which was calculated based on height.

### 2.4. Statistics

Statistical analysis was performed using Prism 10 for Windows version 10.2.3 (403) (GraphPad Software, LLC, La Jolla, CA, USA), Excel 2019 (Microsoft Office 2019), and Analyse-it for Microsoft Excel 6.15.4 (Analyse-it Software, Ltd., Leeds, UK). The Shapiro–Wilk W test was used to determine whether the data followed a normal distribution. When data were not normally distributed, nonparametric statistics were used.

Values are presented as median [first quartile (Q1), third quartile (Q3)] or as mean ± standard deviation and were calculated depending on values distribution.

Significance of differences between pre- and post-treatment test results was determined using the Wilcoxon or Student *t*-test. The significance of the differences between the parameters of the SMA type 2 and type 3 groups was calculated using the Mann–Whitney U test. Statistical significance was set at *p*-value < 0.05. Correlations between test results and demographic and clinical parameters were determined by Spearman’s correlation.

## 3. Results

### 3.1. SMA Type 2 and Type 3 Participants

**Figure 1:**
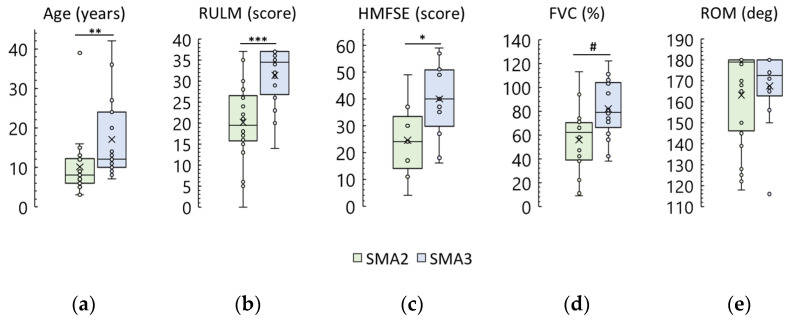
Pre-treatment demographic and clinical data of SMA participants. SMA2 and SMA3—SMA type 2 and 3 participants, respectively. (**a**) Age; (**b**) upper limb function as measured using the Revised Upper Limb Module scale (RULM); (**c**) physical abilities as measured using the Hammersmith Function Motor Scale Expanded (HFMSE); (**d**) pulmonary function as measured using Forced Vital Capacity (FVC); (**e**) knee range of motion (ROM), left and right leg results combined. The crosses are averages, the circles are data points. *, **, and ***—*p* < 0.05, *p* < 0.01, and *p* < 0.001, respectively, as measured using the Mann–Whitney test. #—*p* < 0.05 as measured using Student’s *t*-test.

The groups differed in age (Table 1, Figure 1a). The SMA type 2 group was significantly younger than the SMA type 3 group (*p* = 0.006). There was one adult participant in the SMA type 2 group and six adult participants in the SMA type 3 group (Tables S1 and S2). 

Functionally, the participants were “non-sitters”, “sitters”, or “walkers” [5]. Prior to treatment, participants in the SMA type 2 group were more severe than participants in the SMA type 3 group (Table 2, Figure 1). In the SMA type 2 group, there were predominantly “non-sitters”, and in the SMA type 3 group, there were predominantly “sitters”. There were two “walkers” in the SMA type 3 group. RULM and HFMSE scores were significantly lower in the SMA type 2 group than in the SMA type 3 group (*p* = 0.0004 and *p* = 0.03, respectively). Thirteen SMA type 2 participants and one type 3 participant did not pass this test because they could not sit for 3 s without support on their back or hands (Tables S1 and S2). Pulmonary function, as measured by FVC, was also significantly lower in the SMA type 2 group (*p* = 0.01). Six SMA type 2 participants were unable to complete this test (Table S1). Knee ROM was less in the SMA type 2 group as a trend (*p* = 0.089).

### 3.2. Transcutaneous Spinal Cord Stimulation

Participants tolerated the stimulation well. No adverse events were observed.

The maximal current intensity, averaged over two or three stimulation sites (Tables S3 and S4), was 22 ± 10 mA and 30 [20; 40] mA in the SMA type 2 and type 3 groups, respectively. The difference between the groups was significant (*p* = 0.01); the current intensity used in sessions with SMA type 2 participants was higher.

Training with tSCS lasted for 20 [20; 40] and 30 [25; 45] minutes in the SMA type 2 and 3 groups, respectively. The difference between the groups was non-significant.

### 3.3. Revised Upper Limb Module

**Figure 2:**
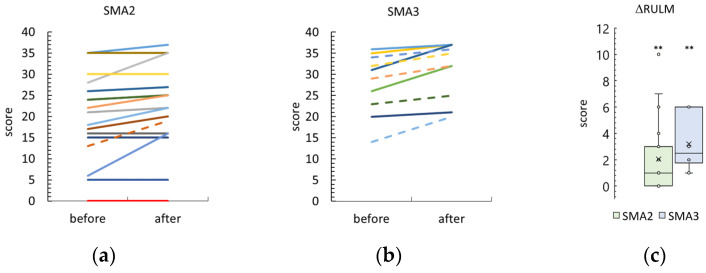
Changes in the Revised Upper Limb Module scale (RULM) score after tSCS course in SMA type 2 and 3 participants ((**a**) and (**b**), respectively). One line—one participant. Dotted lines—adult patients. One SMA type 2 participant and seven SMA type 3 participants showed a maximum score of 37 before and after tSCS sessions. Their results are not shown because the score does not change after the sessions. (**c**)—Difference in RULM score after the sessions in SMA type 2 and type 3 groups. The crosses are averages, the circles are data points. **—*p* < 0.01 as measured using the paired Wilcoxon test.

Nineteen participants in both groups showed an increase in RULM score after the tSCS course (Figure 2 and Figure S1a). One participant in the SMA type 2 group and seven in the SMA type 3 group showed the best score of 37 points before and after the tSCS session; these results were not included. 

The difference between the post- and pre-treatment RULM scores was 1 [0; 3] and 3 [2; 6] points in the SMA type 2 and 3 groups, respectively. Both RULM differences were significantly higher than zero (*p* = 0.001 and *p* = 0.002 in the SMA type 2 and type 3 groups, respectively). The SMA type 3 RULM difference tended to be higher than the SMA type 2 difference (*p* = 0.06).

After tSCS, the RULM score was 22 [16; 29] and 37 [32; 37] in the SMA type 2 and type 3 groups, respectively (Figure S2a). The increase noted in the SMA type 3 group was significant (*p* = 0.0001).

### 3.4. Hammersmith Function Motor Scale Expanded

**Figure 3:**
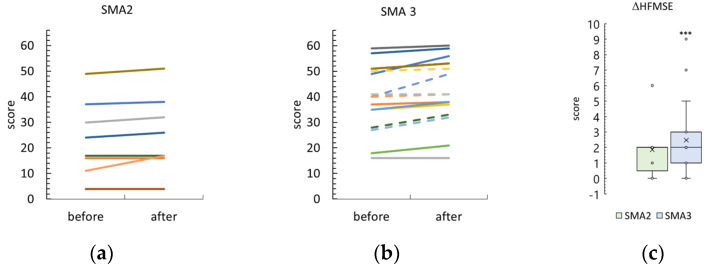
Changes in the Hammersmith Function Motor Scale Expanded (HFMSE) score after tSCS course in SMA type 2 and 3 participants ((**a**) and (**b**), respectively). One line—one participant. Dotted lines—adult patients. (**c**)—Difference in HFMSE score after the sessions in SMA type 2 and type 3 groups. The crosses are averages, the circles are data points. ***—*p* < 0.001 as measured using the paired Wilcoxon test.

Thirteen SMA type 2 participants and one SMA type 3 participant were unable to sit for ~3 s without back and hand support to begin the HMFSE test or took the opportunity to refuse the test due to fatigue. The results of the other participants are shown in Figure 3 and Figure S1b.

The difference between post- and pre-treatment HMFSE scores was 2 [0; 2] and 2 [1; 5] points in the SMA type 2 and 3 groups, respectively. The HMFSE difference was significantly greater than zero in the SMA type 3 group (*p* = 0.0006) and different as a trend in the SMA type 2 group (*p* = 0.06). These HMFSE differences were not significantly different between SMA type 2 and type 3 participants.

After the tSCS sessions, the HMFSE score was 26 [17; 38] and 43 ± 13 in the SMA type 2 and type 3 groups, respectively (Figure S2b). Inequality between the groups after tSCS was significant (*p* = 0.02).

### 3.5. Forced Vital Capacity

**Figure 4:**
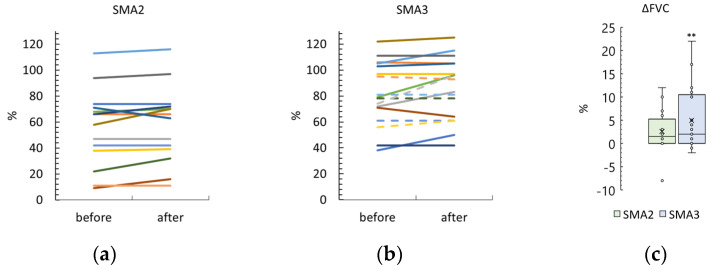
Change in pulmonary function (Forced Vital Capacity, FVC) after tSCS course in SMA type 2 and 3 participants ((**a**) and (**b**), respectively). One line—one participant. Dotted lines—adult patients. (**c**)—Difference in FVC after the sessions in SMA type 2 and type 3 groups. The crosses are averages, the circles are data points. **—*p* < 0.01 as measured using the paired Wilcoxon test.

Six SMA type 2 participants took the opportunity to refuse the breathing test due to fatigue. The results of the others are shown in Figure 4 and Figure S1c.

The difference between post- and pre-treatment FVC was 3 ± 5% and 2 [0; 11]% in the SMA type 2 and 3 groups, respectively. The FVC difference was significantly greater than zero in the SMA type 3 group (*p* = 0.005) and different as a trend in the SMA type 2 group (*p* = 0.08). These differences in FVC after treatment were not significantly different between the SMA type 2 and type 3 groups.

After the tSCS sessions, the FVC scores were 58 ± 29% and 86 ± 24% in the SMA type 2 and type 3 groups, respectively (Figure S2c). The difference between the groups after tSCS was significant (*p* = 0.007).

### 3.6. Knee Range of Motion

**Figure 5:**
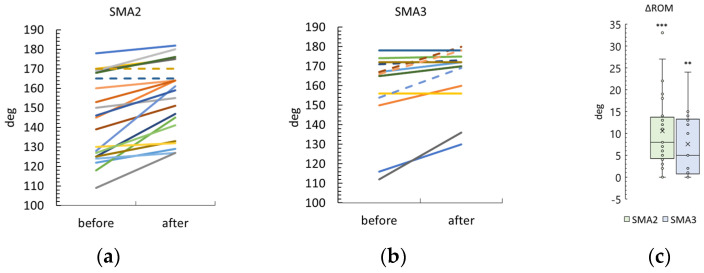
Change in knee range of motion (ROM) after tSCS treatment in SMA type 2 and 3 participants ((**a**) and (**b**), respectively) with pre-treatment knee contractures. Right and left knee ROM results are combined. One line—one participant. Dotted lines—adult patients. (**c**)—Difference in ROM after the course of treatment in SMA type 2 and type 3 groups. The crosses are averages, the circles are data points. *** and **—*p* < 0.0001 and *p* < 0.01, respectively, as measured using the paired Wilcoxon test.

Knee contracture was observed in eleven SMA type 2 participants and eight SMA type 3 participants (Tables S1 and S2). The results for these participants are shown in Figure 5 and Figure S1d. Right and left knee ROM results are combined.

The differences between post- and pre-treatment ROMs were 10 ± 9 degrees and 8 ± 7 degrees in the SMA type 2 and 3 groups, respectively. Both ROM differences were significantly higher than zero (*p* < 0.0001 and *p* = 0.001 in the SMA type 2 and type 3 groups, respectively). The difference in ROM after the stimulation was not significantly different between the SMA type 2 and 3 groups (Figure S2d).

### 3.7. Motor Skills

The opinions of trainers, adult participants, and parents of child participants about significant changes in motor activity and the acquisition of new skills or the restoration of lost skills during the course of tSCS in combination with physical therapy are presented in Tables S4 and S6. In summary, eleven and ten participants in the SMA type 2 and type 3 groups, respectively, gained motor skills after the tSCS course.

The distribution of these motor skills ranged from elementary skills such as lifting the head in the prone and supine positions (participant 6K12) to more complex skills such as walking up and down stairs with one-hand support (instead of two-hand support) (participant 11K34). Participants 1K2, 10K30, 7K14, 8K19, and 9K28 developed skills to control the position of body parts. Participants 21K53, 9K22, 12K42, 7K15, and 4K7 acquired static balance skills. New motor skills related to movement in space were developed by participants 16K50, 3K5, 5K8, 9K26, and 4K7. Participants 8K21 and 15K49 were able to perform coordinated hand movements for self-service.

### 3.8. Functional Status

The functional status of SMA patients determines the trajectory of physical rehabilitation [5]. This study included thirteen non-sitters, twenty-two sitters, and two walkers (Table 2 and Table 3). The results of tSCS treatment were compared between the non-sitter and sitter groups, and the cases of the walkers were analyzed.

**Figure 6:**
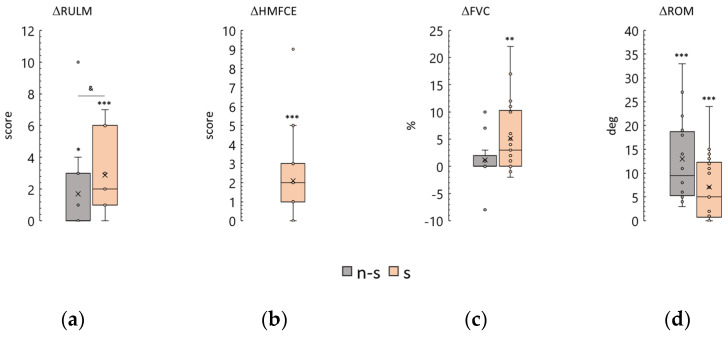
Comparison of differences in outcomes between non-sitters (n-s) and sitters (s). (**a**) Revised Upper Limb Module scale (RULM); (**b**) Hammersmith Function Motor Scale Expanded (HFMSE); (**c**) Forced Vital Capacity (FVC); (**d**) knee range of motion (ROM), combined left and right leg results. The crosses are averages, the circles are data points. *, **, and ***—*p* < 0.05, *p* < 0.01, and *p* < 0.001, respectively, as measured using the paired Wilcoxon test. &—*p* < 0.05 as measured using the Mann-Whitney test.

Expectedly, the results of the start RULM test were significantly greater in sitters than in non-sitters (*p* < 0.0001). The differences in test scores after the course were 0 [0; 3] and 2 [1; 6] points for non-sitters and sitters, respectively. The observed difference is significantly greater than zero in both groups. However, the difference observed in the sitter group is significantly greater than that observed in the non-sitter group (Figure 6a).

Two of the non-sitters were able to pass the HMFSE test. Their results were dramatically lower than those of the sitter group (Table 3). After the course, one non-sitter increased their score from 11 to 17 points, while the other non-sitter’s score remained unchanged. The sitter group demonstrated a change in score of 2 [1; 3], an increase that was statistically significant (Figure 6b).

The FVC test, which measures pulmonary function, revealed that non-sitters had significantly lower results than sitters before the tSCS course (*p* < 0.001) (Table 3). The difference in test results for the non-sitter group was approximately zero (1 ± 5%), while the difference for the sitter group was positive and significant (5 ± 7%) (Figure 6c).

ROM in the knee joint was comparable between the sitter and non-sitter groups prior to treatment, with values of 174 [128; 180] and 174 [165; 180] degrees, respectively. The increase in ROM in contractured knee joints after the tSCS course was 13 ± 9 degrees in non-sitters and 5 [0.8; 12] degrees in sitters (Figure 6d). The increase was positive and significant in both groups.

The two walkers were a 7-year-old boy (11K34) and a 14-year-old adolescent girl (15K48), both with SMA type 3 (Table S2).

Participant 11K34 passed the RULM test with a maximum of 37 points, increased his HMFSE score by 7 from 49 points, and increased his FVC data by 12% from 38%. He had no joint contractures before the course of treatment. His rehabilitation goals were to walk safely on the street and upstairs. After the course of treatment, he managed to cross the street near his house in 15 s while the traffic light was green (before the treatment, this was not possible) and learned to walk upstairs without leaning on the handrail with both hands (Table S6).

Participant 15K48 increased her RULM score by 6 from 31 points, her HMFSE score by 2 from 57 points, and her FVC by 2% from 103%. She had no joint contractures before the course of treatment. After the course, her uninterrupted walking time increased from 15–20 min to 1 h on flat surfaces and small stairs.

Thus, the stimulation course led to an increase in all test parameters in participants from all functional groups. These increases tended to be greater and more significant in the sitter group than in the non-sitter group.

### 3.9. Age and Adults

The efficacy of orphan drugs is restricted to older children and adults with SMA [6,9,10]. A multicenter observation on adult SMA patients treated with nusinersen showed that, although half of the patients experienced a subjective improvement in function, there were no significant objective changes [40]. 

We analyzed the correlation between age and the main tSCS outcomes (Table 4, Figure S3). No association was found between age and differences in outcomes.

Adult participants are indicated by dotted lines in Figure 2, Figure 3, Figure 4 and Figure 5 and are shown in gray in Tables S1–S6.

Adult SMA type 2 participant 14K44, 39 years old, is a sitter with peripheral tetraparesis and has contractures of the knee and ankle joints. After the tSCS course, her knee ROM did not increase and her ankle ROM increased by 3–7 deg. Her RULM score increased by 6 points. She could not be tested for HMFSE score due to the inability to sit without hand or back support. She refused to take part in the breathing study due to fatigue. She did not report significant changes in motor activity or the acquisition of new skills after the course.

**Figure 7:**
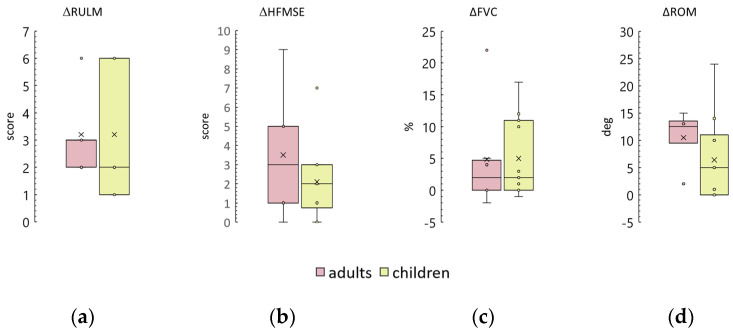
Comparison of differences in outcomes between adults and children in the SMA type 3 group. (**a**) Revised Upper Limb Module scale (RULM); (**b**) Hammersmith Function Motor Scale Expanded (HFMSE); (**c**) Forced Vital Capacity (FVC); (**d**) knee range of motion (ROM), combined left and right leg results. The crosses are averages, the circles are data points.

Six SMA type 3 participants, 20–42 years old, all sitters, were in the SMA type 3 group. In these adult participants, after the tSCS course, RULM increased by 3 [2; 3] points, HMFSE by 3 [1; 5] points, FVC by 2 [0; 5] %, and knee ROM by 12 [9; 13] degrees. The differences tended to increase above zero in the RULM and HMFSE tests (*p* = 0.06 in both) and were insignificantly different from zero in the other tests. These results and those of children in the SMA type 3 group are shown in Figure 7. All differences in outcomes between adults and children in the SMA type 3 group are statistically similar. After the tSCS course, three out of six adult participants and seven out of eleven child participants (50% and 60%, respectively) gained or regained motor skills (Table S6).

### 3.10. Disease Duration

The time between the onset of SMA and the time of specific drug treatment—disease duration—has been shown to be the most important predictor of orphan drug efficacy [11]. We analyzed the relationship between the results of tSCS treatment and disease duration (Table 5 and Figure S4). Correlation coefficients between the differences in the RULM and HMFSE scales and in the FVC test were less than 0.5 and non-significant. We did not detect any effect of disease duration on the outcomes.

### 3.11. Duration of Drug Therapy

Nusinersen treatment progressively increased HMFSE and RULM scores in SMA patients over 15 months, with improvements observed every two months [10]. We analyzed the relationship between tSCS outcomes and the duration of orphan drug treatment (Table 6 and Figure S5). A weak negative correlation, at the trend level, was observed between drug treatment duration and the values of differences in RULM and FVC scores in the SMA type 2 group. Other outcomes did not correlate with treatment duration.

## 4. Discussion

This study evaluated the efficacy and safety of non-invasive spinal cord stimulation for motor rehabilitation in type 2 and type 3 SMA patients treated with orphan drugs. No adverse events were observed in the cohort of thirty-seven participants ranging in age from 3 to 42 years, providing safety data for the use of tSCS in SMA patients.

Early on, we demonstrated a good potential of tSCS in the rehabilitation of SMA patients in a case series report of five pediatric patients aged 6–13 years treated with nusinersen [1]. Now, we increased the number of participants and demonstrated the statistical significance of these changes. The age range of participants in the current study was 3–42 years old, and we have shown that tSCS is as effective in adults as it is in children. The participants of the former study were non-sitters and sitters only, whereas in the current study, other than non-sitters and sitters, some participants were walkers and stimulation led to an improvement in motor function in these participants.

The main finding of the former study was that a 2-week physical therapy protocol combining tSCS with the use of a specific orphan drug treatment led to a significant increase in motor function in both SMA type 2 and type 3 participants. Motor scores increased significantly after tSCS. Twenty-one of thirty-seven participants gained new motor skills or regained lost skills. Respiratory function, which correlates strongly with loss of muscle function [41,42], improved significantly in the SMA type 3 group and trended upward in the SMA type 2 group. Joint contractures, which limit the mobility of SMA patients [5,39], decreased significantly after the course of treatment.

Reliable improvements in motor function should be associated with a concurrent use of stimulation with physical therapy. In controlled studies involving patients with SMA without specific medication support, physical therapy alone has not been shown to alter muscle strength and motor function [43]. Fourteen participants, aged 10–48 years, were randomized to control and exercise groups and followed for 19 months. There were no between-group differences in walking, fatigue, or function at any time point. In another controlled study of the effect of physical therapy on motor function in 35 pediatric SMA type 2 and type 3 patients treated with nusinersen [44], six months of daily physical therapy did not significantly increase HMFSE scores compared to scores of those who did not receive physical therapy (Figure 1 in [44]). Obviously, the physical therapy alone cannot bring about motor improvements in 2 weeks.

### 4.1. Minimal Clinically Important Difference and Stimulation Results

In addition to statistical significance, achieving a minimal clinically important difference (MCID) is meaningful. The MCID is the “smallest change or difference in an outcome measure that is perceived as beneficial and would lead to a change in the patient’s medical management” [45]. The issue of MCID in SMA outcomes is controversial [34,46]. MCIDs for pulmonary and contracture indicators have not been defined for SMA patients. 

#### 4.1.1. RULM and HMFSE Scales

In adult SMA patients, the MCIDs of the RULM and HMFSE scales are 2.9 and 4.3 points, respectively [46]. These values were obtained from 15 and 36 SMA type 2 and type 3 participants, respectively. The authors of that study pointed out the great heterogeneity of the patients’ motor functions. MCID values differed between subgroups (SMA type 2 and type 3 participants, ambulatory and non-ambulatory participants). The authors concluded that further studies are needed to adjust the MCID values.

In a study by Pera et al. [47], parents of 149 SMA type 2 and type 3 patients aged from 17 months to 30 years considered a 1-point increase in HMFSE score to be meaningful. For pediatric SMA patients, the MCID in RULM score has not been established and a change of 2 to 3 points has been recommended as meaningful [34,48].

We observed that twelve out of the twenty-nine participants who did not have a top RULM score of 37 points before the course of treatment increased their RULM score by 2 or more points after the tSCS course (Figure S1a). Eleven out of the twenty-three participants who were tested using the HMFSE increased their HMFSE score by 1 point or more (Figure S1b).

In addition to the magnitude of the outcome differences, the time period over which these positive differences were achieved is important. In a phase 3 clinical trial of nusinersen in eighty-four pediatric SMA type 2 participants (2–9 years old), the average increase in RULM score and HFMSE score was ~1.5 points 3 months after drug treatment (Figure 1 in [10]). In our study, when drug treatment was combined with tSCS and physical therapy for 2 weeks, similar results were obtained. Thus, tSCS accelerates the recovery of drug-induced motor functions.

#### 4.1.2. ROM Results

Minimal hip and knee joint contractures are associated with decreased mobility. Fixed contractures of the knee joint limit the ability to perform functional activities such as rising from a chair, walking up and down stairs, standing, rolling, and comfortable positioning in bed and in a wheelchair [35,39]. In eighty participants with SMA types 2 and 3 (aged 1.1–45.2 years), a decrease in passive knee ROM of 9 degrees or less was shown to correlate with motor deterioration as tested using the HMFSE [35]. We showed that after tSCS, the mean passive knee ROM increased by more than 8 degrees in both SMA groups. These data are associated with increases in motor scale scores.

#### 4.1.3. FVC Results

Spinal muscular atrophy affects the respiratory system [42,49]. In a retrospective study of forty-one untreated SMA type 2 and 3 patients, FVC was strongly correlated with muscle strength [50]; Spearman’s correlation coefficient was 0.78. A natural history study of 170 untreated SMA patients showed a −1.32 to −0.67% reduction in FVC per year [41]. A study in nineteen adult non-ambulatory SMA patients showed that 10–14 months of nusinersen treatment resulted in a stability of FVC outcome measures, with no increase or decrease in this outcome [51]. In our study, FVC increased by 3% as a trend in the SMA type 2 group and significantly by 2% in the type 3 group. Thus, spinal cord stimulation improves lung function when used in combination with orphan drug treatment.

#### 4.1.4. Motor Skill Results

Motor skills were assessed by researchers using the RULM and HMFSE scales. In addition, we interviewed instructors, adult participants, and parents of pediatric participants to learn about the motor skills they felt emerged after the course. These motor skills are not always captured on standard scales; examples of these include moving from the floor to the wheelchair or the ability to wash the face with the right hand without supporting the forearm with the left hand. People who spend a lot of time around patients with SMA pay attention to small changes in motor improvement because new motor skills make it easier to manipulate patients and improve the quality of life of patients and their families. Caregivers of patients with SMA have been found to experience significant burdens, including impaired health-related quality of life, reduced work ability and productivity, and financial stress [52]. 

Twenty-one of thirty-seven participants reported new motor skills after the tSCS course. This is a good result because the rehabilitation of patients with SMA is currently aimed not at improving motor activity but at slowing the process of motor loss and reducing the burden of the disease [5].

### 4.2. Effects of SMA Severity

We found that reliable improvements in motor function were seen in both the SMA type 3 group and in the more severe form of SMA type 2 group. The absolute magnitude of change in all outcomes did not differ between the SMA type 2 and SMA type 3 groups. When comparing the groups in terms of achieving a minimally clinically important difference (MCID) after the course, 58% or more of participants achieved a clinically significant rate after the course (Figure S1). Of the 19 participants with type 2 SMA who had less than the maximum 37 RULM scores, 11 achieved an MCID post-treatment. Similarly, in the group of participants with type 3 SMA, 8 out of 10 participants achieved an MCID after the course. Of the seven participants with SMA type 2 who were able to complete the HFMSE, five achieved an MCID on this scale after the course. Similarly, in the SMA type 3 group, 13 out of 16 participants achieved an MCID. 

However, disease severity is important in predicting the course of tSCS. We analyzed the magnitude of change in scores as a function of the functional severity of the participants. After one session of stimulation, severe non-sitters had significantly less change than sitters with less motor impairment (Figure 6a,c). Thus, the magnitude of positive changes in motor function after the course is smaller in patients with less motor ability.

### 4.3. Effects of Age, Disease Duration, and Medication Duration

The dysfunction and degeneration of α-motor neurons in the spinal cord occur with age in the natural history of SMA patients [3]. Orphan drug treatment is more effective if started early [6,53]. Disease duration is closely related to age, and similar to age, the effects of SMA-specific drugs are greater in patients with shorter disease duration [11]. Therefore, we predicted that stimulation would be more effective in pediatric patients and in patients with shorter disease duration than in adults and in patients with longer disease duration. We analyzed the correlation between age, disease duration, duration of medical therapy, and the magnitude of changes in all parameters controlled in this study. No association was found between age or either duration and differences in outcomes. This is unexpected. It is possible that the relationship may change as the number of patients in this study increases. Perhaps a complex of parameters determines the dependence of stimulation results. There is evidence that disease duration and drug therapy duration in combination significantly alter motor unit response to nusinersen over time in children with SMA [26]. We were not able to verify such a dependency in the data we obtained, as most of the data analyzed were not normally distributed.

Using stimulation to rehabilitate patients with SMA, we hypothesized that activation of spinal motor networks and modulation of motoneuron activity underlie the therapeutic effect [14,18]. We attributed this mechanism to the expected dependence of the magnitude of the stimulation effect on the predicted number of preserved motoneurons. It is possible that direct stimulation of 1a afferents may have a therapeutic effect too. It has been shown in animal models that motor neuron dysfunction in SMA begins with a decrease in excitatory input from primary afferents [3,54]. The lack of dependence of the effect of the stimulation course on the patient’s age, duration of symptoms, and duration of orphan therapy suggests a complex mechanism of motor improvement, one component of which may be the excitation of primary motor neuron afferents.

It is possible that stimulation not only activates preserved motoneurons, but also “prosthetizes” absent excitatory afferents, leading to the rapid recovery of motor functions and to the independence of this effect from the duration of the disease and the duration of specific drug treatment.

### 4.4. Limitations and Future Directions

The lack of a control group is a dramatic limitation in many studies, but not in our study. Daily physical therapy during 19 months did not modify motor outcomes in SMA patients without orphan drug treatment [43], nor did physical therapy administered during 6 months do so in patients treated with nusinersen [44]. Clearly, two weeks of physical therapy alone would not improve motor function in SMA patients. For rational and ethical reasons, we did not include a control group in our studies. The relatively small cohort with heterogeneous phenotypes (SMA type 2 and type 3 non-sitters, sitters, and walkers with a wide range of disease durations and drug therapies) may complicate the interpretation of our data. Problems with interpretation indicate the need for further studies. We need to increase the number of mixed-age group to verify the probable dependence of tSCS course on the combination of disease duration and SMA-specific therapy duration. Another direction is the electrophysiological study of the probable activation of excitatory afferents of motor neurons during tSCS in SMA patients.

## 5. Conclusions

A two-week course of stimulation combined with physical therapy in patients taking nusinersen and other orphan drugs resulted in significant increases in motor function, improved respiratory function, and decreased contracture in both type 2 and type 3 SMA participants in this group. The magnitude of functional changes did not vary with patient age, disease duration, and drug therapy duration. Further studies are needed to elucidate the reasons for the beneficial effects of spinal cord electrical stimulation on SMA patients.

## Figures and Tables

**Table 1:** Demographic and clinical parameters of the studied SMA groups.

Group	N	Sex (m/f)	Age (years)	Orphan Drug (n)		
				Nusinersen	Risdiplam	OA
SMA2	20	11/9	8 [6; 13] ^1^	13	6	1
SMA3	17	10/7	12 [10; 24]	17	-	-

^1^ median [first quartile, Q1; third quartile, Q3]. m—male; f—female; OA—onasemnogene abeparvovec.

**Table 2:** Clinical parameters of the studied SMA groups.

Group	Funct. Status (n)			RULM	HFMSE	FVC
	N-S	S	W	(Score)		(%)
SMA2	12	8	-	20 ± 10 ^1^ (20) ^2^	24 [11; 37] ^3^ (7)	56 ± 30 (14)
SMA3	1	14	2	35 [28; 37] (17)	40 ± 13 (16)	82 ± 24 (17)

^1^ mean ± standard deviation. ^2^ (n, number of the tested participants). ^3^ median [first quartile, Q1; third quartile, Q3]. Funct. status—functional status; N-S—non-sitters; S—sitters; W—walkers; RULM—Revised Upper Limb Module; HFMSE—Hammersmith Function Motor Scale Expanded; FVC—Forced Vital Capacity.

**Table 3:** Demographic and clinical parameters of participants related to functional status.

Funct. Status	n	Age	RULM	HFMSE	FVC
		(Years)	(Score)		(%)
N-S	13	9.5 ± 4.1 ^1^	16 ± 8	4; 11 (2) ^3^	47 ± 27 (11)
S	22	11.5 [8; 24] ^2^	35 [26; 37]	36 ± 13 (19)	84 ± 21 (18)
W	2	7; 14	31; 37	49; 57	38; 103

^1^ mean ± standard deviation. ^2^ median [first quartile, Q1; third quartile, Q3]. ^3^ Number of the participants who have passed the test. N-S—non-sitters; S—sitters; W—walkers; RULM—Revised Upper Limb Module; HFMSE—Hammersmith Function Motor Scale Expanded; FVC—Forced Vital Capacity.

**Table 4:** Spearman’s correlation coefficient (r), *p*-value, and number of pairs (n) for age and tSCS outcome differences.

Group	ΔRULM			ΔHFMSE			ΔFVC		
	r	*p*	n	r	*p*	n	r	*p*	n
SMA2	0.26	0.27	9	0.32	0.48	7	0.11	0.71	14
SMA3	0.49	0.16	10	0.09	0.75	16	−0.09	0.74	17

**Table 5:** Spearman’s correlation coefficient (r), *p*-value, and number of pairs (n) for disease duration and tSCS outcome differences.

Group	ΔRULM			ΔHFMSE			ΔFVC		
	r	*p*	n	r	*p*	n	r	*p*	n
SMA2	0.41	0.11	16	0.15	0.83	5	0.16	0.59	13
SMA3	0.37	0.41	7	0.26	0.40	12	0.39	0.19	13

**Table 6:** Spearman’s correlation coefficient (r), *p*-value, and number of pairs (n) for duration of drug therapy and tSCS outcome differences.

Group	ΔRULM			ΔHFMSE			ΔFVC		
	r	*p*	n	r	*p*	n	r	*p*	n
SMA2	−0.44	0.08	17	0.62	0.24	6	−0.47	0.09	14
SMA3	−0.15	0.72	8	0.06	0.84	14	−0.01	0.96	15

## Data Availability

The datasets generated and/or analyzed during the current study are available from the corresponding author on reasonable request.

## Supplementary Materials

The following supporting information can be downloaded at: https://www.mdpi.com/article/10.3390/biomedicines12061162/s1, Figure S1: Frequency distribution of the differenced in RULM, HFMSE, FVC, and ROM results before and after tSCS sessions in SMA type 2 and type 3 groups; Figure S2: Clinical data of SMA participants after stimulation course; Figure S3: Difference in RULM, HFMSE, and FVC results before and after tSCS sessions versus age; Figure S4: Difference in RULM, HFMSE, and FVC results before and after tSCS sessions versus disease duration; Figure S5: Difference in RULM, HFMSE, and FVC results before and after tSCS sessions versus duration of drug therapy; Table S1: Demographics and clinical parameters of SMA type 2 participants; Table S2: Demographics and clinical parameters of SMA type 3 participants; Table S3: Spinal stimulation parameters of SMA type 2 participants; Table S4: Spinal stimulation parameters of SMA type 3 participants; Table S5: New motor skills of participants with SMA type 2 after the tSCS course; Table S6: New motor skills of participants with SMA type 3 after the tSCS course.

## Acronyms and Abbreviations

FVC	Forced vital capacity
HFMSE	Hammersmith Function Motor Scale Expanded scale
N-S	Non-sitters
OA	Onasemnogene abeparvovec
ROM	Range of Movements of joints with contracture
RULM	Revised Upper Limb Module scale
S	Sitters
tSCS	transcutaneous Spinal Cord Stimulation
W	Walkers
