# Supplementary material for: Non-Invasive Spinal Cord Stimulation for Motor Rehabilitation of Patients with Spinal Muscular Atrophy Treated with Orphan Drugs
Source: Biomedicines. 2024 May 24;12(6):1162. doi: 10.3390/biomedicines12061162 (PMC11200420; doi:10.3390/biomedicines12061162)
Supplement: Supplementary file 1 [file biomedicines-12-01162-s001.zip › biomedicines-2991867-supplementary.pdf]

Acronyms and Abbreviations

F	frequency
lvl	level of the spinal cord
Nus	Nusinersen
Risd	Risdiplam
Treatm	Treatment duration

Table S1. Demographics and clinical parameters of SMA type 2 participants

Adult participant is marked in gray

Participants	Sex	Age, years	Orphan drug				RULM, score	HFMSE, score	FVC, %	Knee ROM, deg		Functional status		
			Nus	Risd	OA	Treatment, months				Right	Left	N-S	S	W
21K53	M	3	Y			21	37	37					Y	
6K12	M	3	Y			24	16					Y		
8K18	M	5	Y			--[1]	21	30					Y	
15K49	M	5			Y	30	16			178	168	Y		
1K1	M	6	Y			--	35						Y	
10K30	F	6	Y			31	24		74	145	128	Y		
5K10	M	6		Y		26	15		66			Y		
1K2	F	7		Y		8	17	4	47			Y		
15K47	M	7	Y			26	16		38	150	160	Y		
9K22	F	8	Y			34	35	49	113				Y	
16K50	M	8	Y			27	26	24	68	170	169		Y	
3K5	F	9		Y		17	24	17	66	122	130		Y	
6K11	F	9	Y			13	6		9	118	124	Y		
9K23	M	12	Y			19	22	11	94	125	127	Y		

5K8	M	12		Y		5	28		58	139	146		Y	
8K20	F	13		Y		34	30		71			Y		
9K24	F	13	Y			21	18		22			Y		
7K14	F	15	Y			16	0		42	169	153	Y		
8K19	M	16		Y		31	5		11	125	109	Y		
14K44	F	39	Y			36	13			165	170		Y	

[1] Medical records of initial treatment not provided

Table S2. Demographics and clinical parameters of SMA type 3 participants
Adult participants are marked in gray

Participants	Sex	Age, years	Orphan drug				RULM, score	HFMSE, score	FVC, %	Knee ROM, deg		Functional status		
			Nus	Risd	OA	Treatment, months				Right	Left	N-S	S	W
11K34	M	7	Y			57	37	49	38					Y
20K51	M	8	Y			47	37	37	106				Y	
12K43	M	9	Y			36	37	16	72	116	112		Y	
9K26	M	10	Y			22	35	40	97	150			Y	
12K42	F	10	Y			38	36	35	105	165			Y	
7K15	F	11	Y			--[1]	26	18	79	156	172		Y	
11K39	F	11	Y			23	20		42			Y		
9K27	M	12	Y			32	37	51	71	167	178		Y	
2K3	M	12	Y			22	37	59	111				Y	
11K35	M	13	Y			20	37	51	122	174	165		Y	
15K48	F	14	Y			35	31	57	103					Y
9K28	M	20	Y			20	23	28	78				Y	
10K32	F	24	Y			16	34	40	61	171	154		Y	
4K7	M	24	Y			22	29	40	95	167	166		Y	
9K25	F	27	Y			13	37	41	74				Y	
11K37	F	36	Y			21	32	50	56				Y	
8K21	M	42	Y			--	14	27	81				Y	

[1] Medical records of initial treatment not provided

Table S3. Spinal stimulation parameters of SMA type 2 participants
Adult participant is marked in gray

Participants	Cervical lvl		Thoracolumbar lvl		Sacral lvl		tSCS duration[2], min	Therapy duration, days
	Current[1], mA	F, Hz	Current[1], mA	F, Hz	Current[1], mA	F, Hz		
21K53	10	20	10	20			20	12
6K12	22	30	22	30	22	30	30	12
8K18	18	30					40	12
15K49	12	30	12	30			40	12
1K1			30	20	30	20	20	12
10K30			18	30	18	35	35	12
5K10	20	30	20	30			20	10
1K2	7	30	7	30			45	12
15K47	45	30			39	30	40	10
9K22	25	30					20	12
16K50	30	30	30	30			20	12
3K5	10	30					20	12
6K11	45	30	27	30			10	12
9K23	20	30					20	12
5K8	30	30	30	30			50	10
8K20	25	30	25	30			35	12
9K24	12	30					20	10
7K14	30	30	30	30			15	12
8K19	30	30	30	30			50	12
14K44	12	20	12	20			15	12

[1] maximal current intensity during tSCS course; [2] estimated duration per day excluding breaks to relax participants and change positions for the next therapeutic exercise.

Table S4. Spinal stimulation parameters of SMA type 3 participants
Adult participants are marked in gray

Participants	Cervical lvl		Thoracolumbar lvl		Sacral lvl		tSCS duration[2], min	Therapy duration, days
	Current[1], mA	F, Hz	Current[1], mA	F, Hz	Current[1], mA	F, Hz		
11K34	15	30	15	30			30	12
20K51	20	20	20	20			50	12
12K43	23	30	18	30			50	10
9K26	32	30	42	30			30	12
12K42	65	30			65	30	35	12
7K15	60	30					20	12
11K36	30	30	30	30			45	8[3]
9K27	40	30	40	30			30	12
2K3	30	30					30	12
11K35	40	30	40	30			20	12
15K48	15	30	49	30			40	12
9K28	15	30	20	30			30	12
10K32			55	30			35	13
4K7	45	30	27	30	20	20	40	12
9K25	20	30	20	30			30	12
11K37	15	30	25	30			20	12
8K21	30	30	30	30			50	10

lvl – level; [1] maximal current intensity during tSCS course; [2] estimated duration per day excluding breaks to relax participants and change positions for the next therapeutic exercise; [3] the caregivers of patient 11K36 reported after the seventh stimulation that for personal reasons they could no longer bring the participant in 2 days later, it was decided to perform an additional (eighth) day of stimulation and a final test.

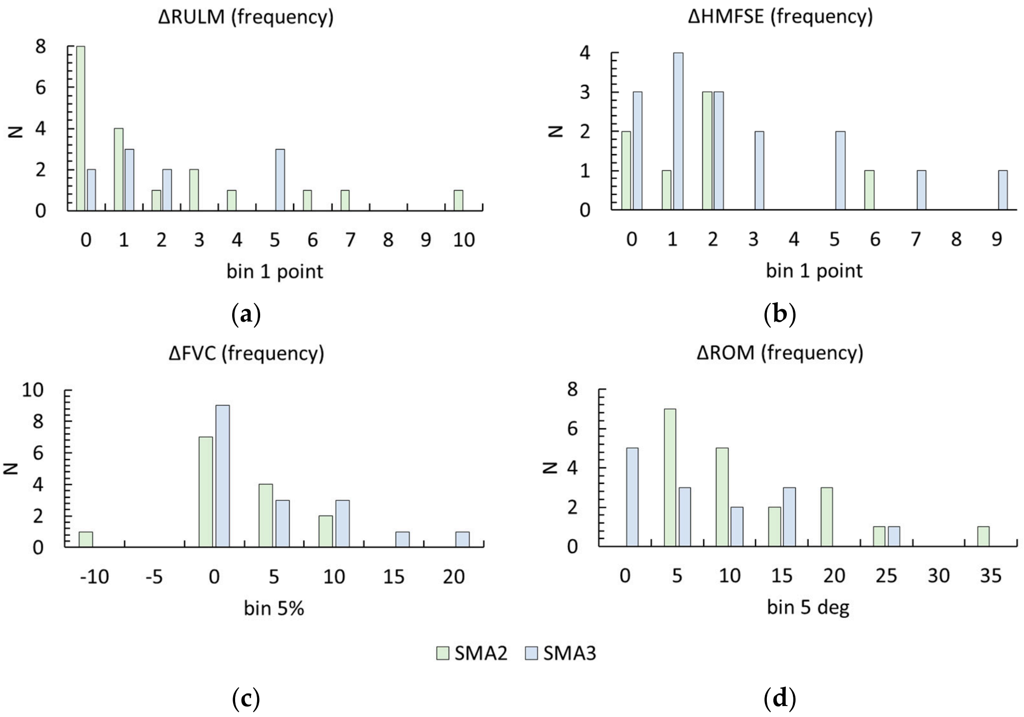

Figure S1. Frequency distribution of the difference in RULM (**a**), HFMSE (**b**), FVC (**c**), and ROM, left and right leg results combined (**d**) before and after tSCS sessions in SMA type 2 and type 3 groups (SMA2 and SMA, respectively). Bin is 1 point in (**a**) and (**b**) and 5 percent and degrees in (**c**) and (**d**), respectively.

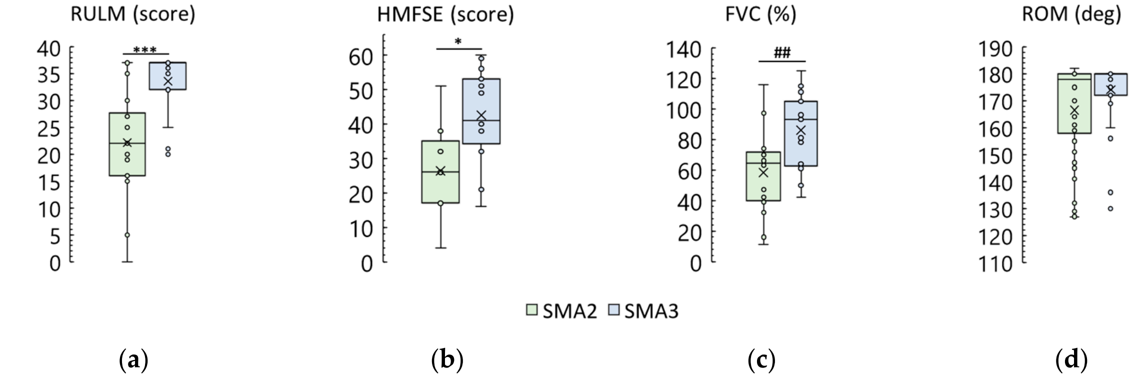

Figure S2. Clinical data of the SMA participants after stimulation course. SMA2 and SMA3 – SMA type 2 and 3 participants, respectively. (**a**) RULM; (**b**) HFMSE; (**c**) FVC; (**d**) ROM, left and right leg results combined. * and *** - $p<0.05$ and $p<0.001$, respectively, by Mann-Whitney test. ## - $p<0.01$ by Student test.

Table S5. New motor skills of participants with SMA type 2 after the tSCS training course
Adult participant is marked in gray

Participants	New motor skills
21K53	Standing unsupported up to 1 minute
6K12	Head elevation in prone position. Head elevation from pillow in supine position
8K18	
15K49	When eating, uses a spoon
1K1	
10K30	Moving from supine to sitting with hands
5K10	
1K2	Supine, legs vertical, up to 15 seconds
15K47	
9K22	Standing unsupported up to 2,5 minutes
16K50	Moves from wheelchair to couch without support
3K5	Sitting on the floor, moves forward 2-3 meters using hands and legs
6K11	
9K23	
5K8	Moves from the floor to wheelchair without support
8K20	
9K24	
7K14	Rolling from the supine to the lateral position
8K19	Raises and holds knees in supine position
14K44	

Table S6. New motor skills of participants with SMA type 3 after the tSCS training course
Adult participants are marked in gray

Participants	New motor skills
11K34	Use one hand (instead of two) to walk up and down stairs
20K51	
12K43	
9K26	Moves from all fours to wheelchair without support
12K42	Standing unsupported and walking 2-3 meters supported
7K15	Standing on all fours up to half a minute
11K39	Movement from supine to right side position
9K27	Pick up and move 100-gram weight
2K3	
11K35	Ability to extend from standing with hands on knees to standing upright
15K48	
9K28	Movements from supine to prone position and from prone position to supine position
10K32	
4K7	Transition from sitting on the floor to kneeling on the floor, kneeling on both knees
9K25	
11K37	
8K21	The ability to wash the face with the right hand without supporting the forearm with the left hand has returned

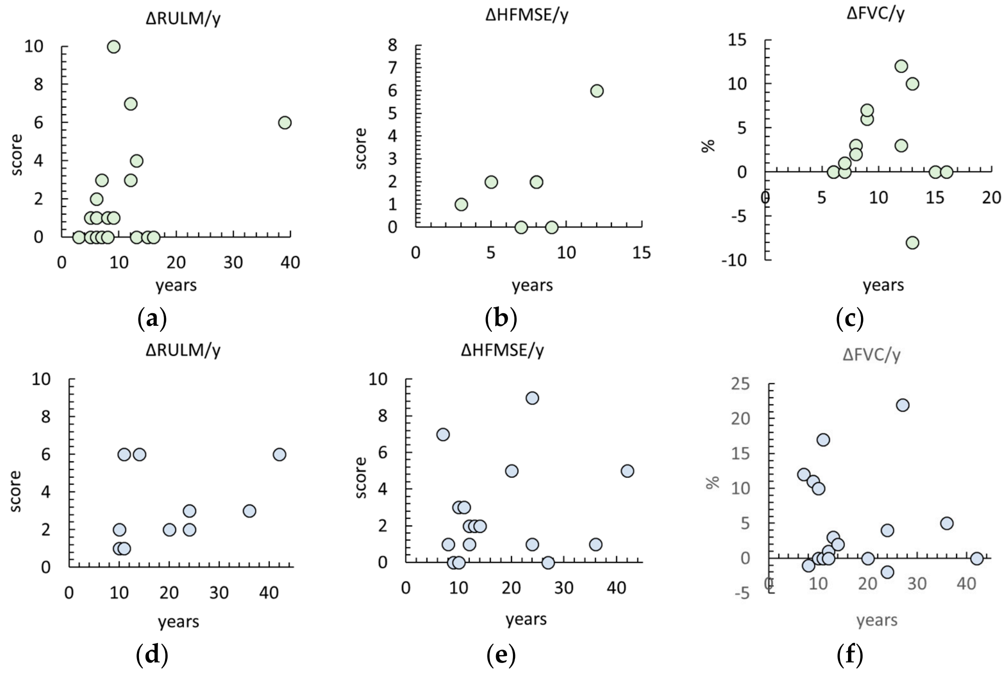

Figure S3. Difference in RULM, HFMSE and FVC before and after tSCS sessions versus age. (**a**) - (**c**) - SMA type 2 group; (**d**) - (**f**) - SMA type 3 group.

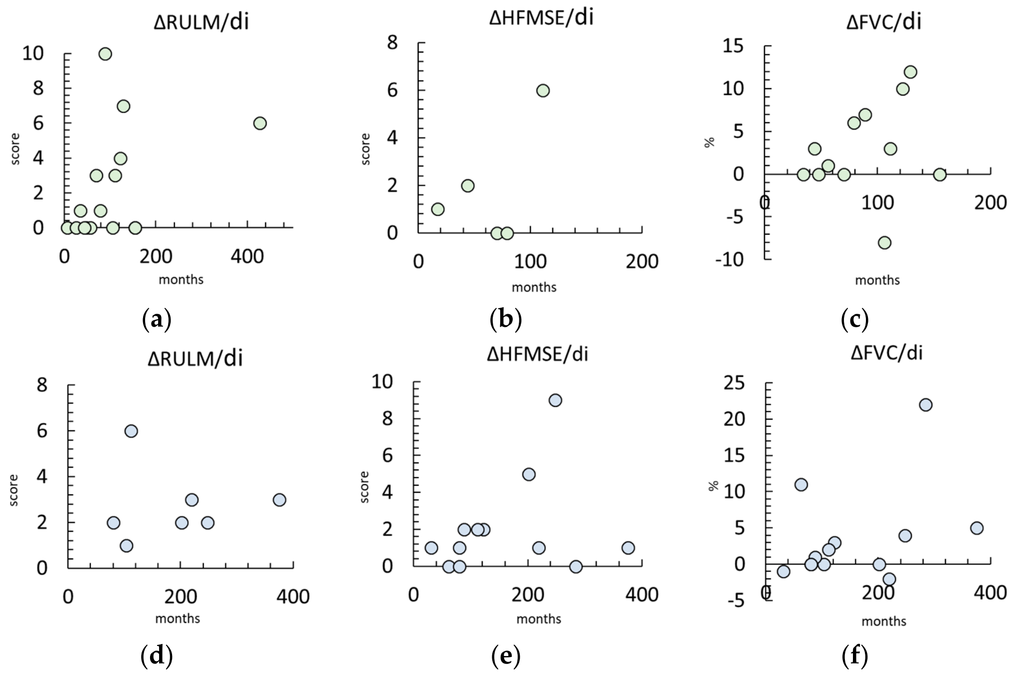

Figure S4. Difference in RULM, HFMSE and FVC before and after tSCS sessions versus disease duration. (a) - (c) - SMA type 2 group; (d) - (f) - SMA type 3 group.

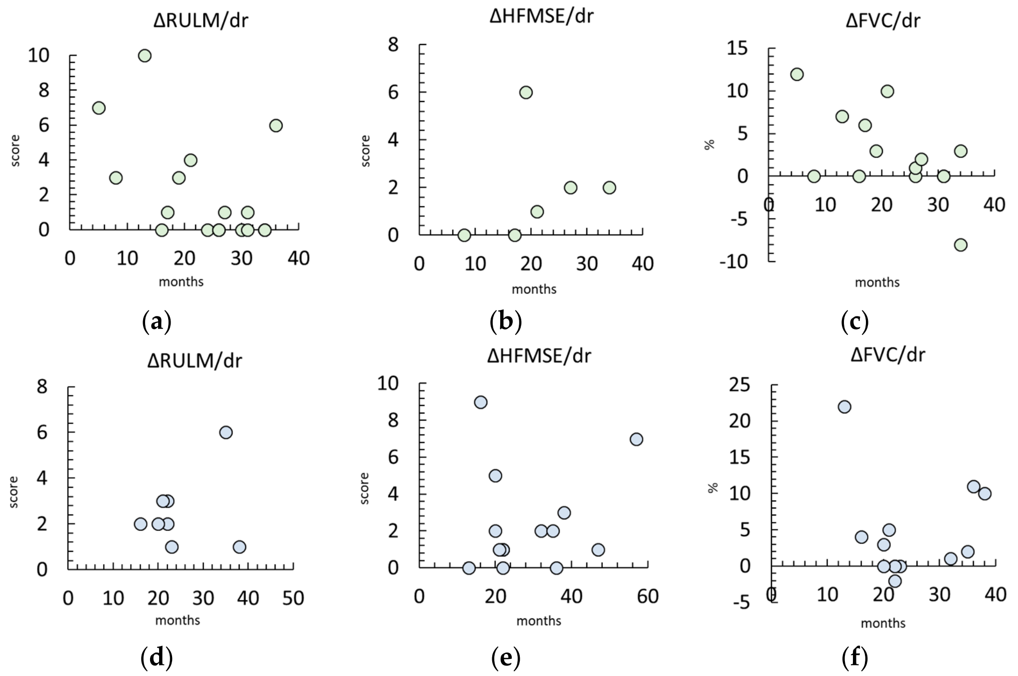

Figure S5. Difference in RULM, HFMSE and FVC before and after tSCS sessions versus duration of drug therapy. (a) - (c) - SMA type 2 group; (d) - (f) - SMA type 3 group.
